# A Green Desulfurization Technique: Utilization of Flue Gas SO_2_ to Produce H_2_ via a Photoelectrochemical Process Based on Mo-Doped BiVO_4_

**DOI:** 10.3389/fchem.2017.00114

**Published:** 2017-12-12

**Authors:** Jin Han, Kejian Li, Hanyun Cheng, Liwu Zhang

**Affiliations:** Shanghai Key Laboratory of Atmospheric Particle Pollution and Prevention, Department of Environmental Science and Engineering, Fudan University, Shanghai, China

**Keywords:** hydrogen, sulfur dioxide, solar energy, Photoelectrochemical (PEC), Mo-doped BiVO_4_

## Abstract

A green photoelectrochemical (PEC) process with simultaneous SO_2_ removal and H_2_ production has attracted an increasing attention. The proposed process uses flue gas SO_2_ to improve H_2_ production. The improvement of the efficiency of this process is necessary before it can become industrial viable. Herein, we reported a Mo modified BiVO_4_ photocatalysts for a simultaneous SO_2_ removal and H_2_ production. And the PEC performance could be significantly improved with doping and flue gas removal. The evolution rate of H_2_ and removal of SO_2_ could be enhanced by almost three times after Mo doping as compared with pristine BiVO_4_. The enhanced H_2_ production and SO_2_ removal is attributed to the improved bulk charge carrier transportation after Mo doping, and greatly enhanced oxidation reaction kinetics on the photoanode due to the formation of ${\text{SO}}_{3}^{2-}$ after SO_2_ absorption by the electrolyte. Due to the utilization of SO_2_ to improve the production of H_2_, the proposed PEC process may become a profitable desulfurization technique.

## Introduction

Sulfur dioxide (SO_2_), as one of the acid gases, could transform into some atmospheric products (e.g., sulfate, sulfuric acid aerosol) through the chemical process in the atmosphere. It and its atmospheric products are detrimental to human health, and cause lots of environmental problems such as smog formation, acid deposition, and degradation of visibility. As is well-known, the SO_2_ mainly enters the atmosphere through the anthropogenic processes, e.g., combustion and release of petroleum, fossil fuel, etc. (Lelieveld and Heintzenberg, 1992; McDonald-Buller et al., 2016). Till now, to remove SO_2_ released from the burning of fossil fuels, several effective methods has been developed (Srinivasan and Grutzeck, 1999; Bashikova et al., 2001; Xia et al., 2011; Kaplan et al., 2013; Yang et al., 2013). Among these technologies, Wet Flue Gas Desulfurization (WFGD) has been one of the state-of-the-art technologies for SO_2_ removal with high efficiency, simple equipment and obtaining multi-useful byproducts (Yang et al., 2012; Lu et al., 2013). Unfortunately, there are some inevitable drawbacks with the WFGD process: for example, the oxidation process energy of ${\text{SO}}_{3}^{2-}$ to final ${\text{SO}}_{4}^{2-}$ is wasted and it is not consistent with the sustainable principles. In order to solve this problem, our group proposed a solar-to-H_2_ energy conversion with SO_2_ removal simultaneously via a solar water splitting process for the first time (Han et al., 2017). Since the oxidation of ${\text{SO}}_{3}^{2-}$ (formed during desulfurization process) needs much lower activation energy and 2 electrons than that of water, acted as a sacrificing reagent during the process, thus it could significantly improve the evolution rate of H_2_. This method not only utilizes the waste energy during the desulfurization process, also realizes the energy production by using air pollutants, which could achieve the zero release of SO_2_.

Though we have reported a photoresponse semiconductor (BiVO_4_) for H_2_ generation with SO_2_ removal, but it is still a challenge to facilitate the performance due to the intrinsic low mobility of photogenerated charges of BiVO_4_. Lots of methods have been proposed to solve this problem, such as controlling the morphology (McDonald and Choi, 2012; Kim and Choi, 2014; Zhou et al., 2014), metal and nonmetal doping (Jo et al., 2012; Chen et al., 2015; Huang H. et al., 2015; Huang H. W. et al., 2015; Huang et al., 2017), forming heterojunctions (Hong et al., 2011; Luo et al., 2011; Seabold and Choi, 2012; Zhang et al., 2016). These modifications have improved the properties of BiVO_4_ greatly by reducing the band-gap energy or improving the charge carrier transport. It has been reported that Mo^6+^ ion substitute the V site in monoclinic sheelite BiVO_4_ could improve the photoinduced carriers, which could facilitate the oxidation while in water splitting reaction system theoretically and experimentally (Luo et al., 2011, 2013; Parmar et al., 2012; Ding et al., 2014; Park et al., 2014; Seabold et al., 2014; Zhou et al., 2014; Jiang et al., 2015, 2017; Kuang et al., 2016; Nair et al., 2016; Pattengale and Huang, 2016). However, Mo-doped BiVO_4_ has not been studied as photoanode for flue gas SO_2_ removal. Here, we prepared Mo-doped BiVO_4_ for enhancing H_2_ generation with simultaneous SO_2_ removal. Moreover, the importance of the amount of Mo-dopants on the performance of BiVO_4_ with SO_2_ removal was also studied. Besides we prepared a series of Mo-doped BiVO_4_ films with different content. The structure characterizations of the obtained films are investigated by XRD, SEM, Raman, UV-vis. Furthermore, we studied the performance of H_2_ generation and efficiency of SO_2_ removal.

## Experiment

### Synthesis of the catalysts

In this work, the deionized water (DI water) was used throughout the whole experiment, and all the chemical reagents are analytical grade and used without any further purification.

F-doped SnO_2_ coated glass (FTO) were purchased from China Southern Glass Co. Ltd, and the FTO glasses were sonicated by immersing in acetone, ethanol and DI water for removing the impurities on the surface of the glass. For comparison, the pristine BiVO_4_ was also prepared. All of the electrodes were synthesized by drop-coating method. The precursor solution was dropped onto the conducting side of FTO, followed by annealing in air. The precursor solutions were synthesized by the following procedure (Zhang et al., 2014): diethylene-etriaminepentaacetic acid (DTPA) and ammonia in water (13.0 mol L^−1^) were added into hot deionized water. After dissolution, the stoichiometric Bi(NO_3_)_3_•5H_2_O, V_2_O_5_ powder and moderate ammonium molybdate tetrahydrate (H_24_Mo_7_N_6_O_24_·4H_2_O) were added into sequence as listed. The resulted mixture was stirred and heated for an hour to promote the dissolution and reaction (complexation of Bi^3+^, V^5+^, and Mo^6+^ with DTPA) until the mixture turned into a transparent solution. Here, we prepared three different samples with the content of Mo ranging from 1, 3, to 5. The amounts of doped Mo were 1 atom%, 3 atom%, 5 atom%, and were denoted as BiVO_4_(Mo-1), BiVO_4_(Mo-3), BiVO_4_(Mo-5), respectively. Then, 40 μl prepared solutions were dropped onto the conducting side of FTO (1 × 2 cm) respectively. After dried at 60° C in oven, the films were annealed at 500° C for 3 h with a ramping rate of 2° C min^−1^ in air. The above process was repeated by three times for the synthesis of the electrode.

### Characterization of the samples

The crystal structures of the as-prepared samples were determined using an X-Ray Diffraction (XRD) with Cu Kα radiation ranging from 10 to 60°. The crystallite sizes of the samples were calculated using the Scherrer formula:

(1) $$\begin{array}{l} D=\frac{K\lambda }{\beta \text{cos}\theta } \end{array}$$

Where *D* is the average crystallite size (nm), λ is the wavelength of the X-ray radiation (0.154 nm), K is the shape factor (0.9), β is the peak width at half-maximum height, corrected for instrumental broadening, and 2θ = 28.7°. The micromorphology and the microstructure of the samples were determined by using field emission scanning electron microscopy (FE-SEM, Hitachi S-4800, Japan). UV-vis transmission spectra of the as-prepared catalysts were measured using a UV-Vis spectrophotometer (SHIMADZU UV-2600) with an integrating sphere attachment. BaSO_4_ used as a standard. Raman spectra were recorded with a Raman spectrometer (HORIBA, X-plo RA Plus), a green laser (532 nm) were used as excitation sources.

### PEC measurement

The apparatus for the PEC tests was a gastight photoreactor. The PEC performance were conducted with a typical three-electrode configuration by using a potentiostat (CHI 660E, Shanghai Chenhua Co. Lid. China). The synthesized pure BiVO_4_ or Mo-doped BiVO_4_ electrode was used as working electrode, Pt wire was used as counter electrode and Hg/HgO electrode was used as reference electrode. The electrolyte solutions for the PEC tests were prepared by absorbing SO_2_ gas with NaOH solutions of certain concentration (detailed in Table 1). We bubbled SO_2_ of concentration is 1,000 ppm successively to NaOH aqueous with the flow rate of 200 ml min^−1^ to form Na_2_SO_3_ with a specific concentration. Eventually, the obtained electrolytes for PEC tests were 0.1 M NaOH−0.025 M Na_2_SO_3_ [denoted as NaOH(aq)+SO_2_(g)-1], 0.1 M NaOH-0.05 M Na_2_SO_3_ [denoted as NaOH(aq)+SO_2_(g)-2], 0.1 M NaOH-0.075 M Na_2_SO_3_ [denoted as NaOH(aq)+SO_2_(g)-3]. For comparison, the NaOH (aq, 0.1 M) solution was also prepared. The PEC tests were measured under illumination by using a 300 W Xe lamp solar simulator with AM 1.5 G filter (100 mW cm^−2^) from the back side of the working electrode, as well as in dark conditions. Liner Sweep Voltammetry (LSV) was measured with the sweep rate of 10 mV s^−1^.

**Table 1:** The detailed information of electrolyte and SO_2_ absorbing efficiency.

**Items**	**NaOH(aq)-SO_2_(g)-1**	**NaOH(aq)-SO_2_(g)-2**	**NaOH(aq)-SO_2_(g)-3**
Concentration of NaOH/M	0.150	0.200	0.250
Concentration of SO_2_/ppm	1,000	1,000	1,000
SO_2_ flow rate/ml min^−1^	200	200	200
SO_2_ inletting time/min	112	224	336
Resulted concentration of ${\text{SO}}_{3}^{2-}$/M	~0.025	~0.050	~0.075
SO_2_ absorbing efficiency/%	~99	~98	~98

The structures of prepared BiVO_4_ and Mo-doped BiVO_4_ bulk were optimized by using the CASTEP code (Ding et al., 2014). The primitive cell of pure monoclinic sheelite BiVO_4_ was relaxed using 400 eV energy cut off for the plane-wave expansion. The structural model of Mo-doped monoclinic sheelite BiVO_4_ was built by substituting one V atom in a relaxed (2 × 1 × 2) supercell of monoclinic sheelite BiVO_4_ with one Mo atom.

### Hydrogen evolution

The reactor for hydrogen evolution experiments is identical with the apparatus used for PEC tests, which use a two-electrode configuration. The electrolyte was NaOH solution bubbled with SO_2_ gas. The experiments were conducted under illumination using a 300 W Xe lamp with AM 1.5 G filter (100 mW cm^−2^) from the back side of the photoanodes in a 150 ml reactor with 100 ml electrolytes filled in and the external bias was 1.6 V. The amount of H_2_ was analyzed by gas chromatography using a thermal conduction detector (TCD) once an hour. The pH values of the solution during the PEC tests were detected by Ohaus (STARTER 2100). The theoretical evolution rate of H_2_ is calculated according to the following equation:

(2) $$\begin{array}{l} v\;=\frac{I\times t}{Z\times F\times A\times 3600} \end{array}$$

Where, *v* indicates the evolution rate of H_2_ (mol cm^−2^ h^−1^); *I* indicates the average current (A); *t* indicates the time (s); *Z* indicates the transferred electron number (1); *F* is the Faraday's constant (96,500 C mol^−1^); *A* is the area of the film (cm^2^).

The equation for the calculation of Faradaic efficiency is:

(3) $$\begin{array}{l} Faradaic\;efficiency\;\left(\%\right)=\frac{m\times n\times F}{I\times t}\times 100\% \end{array}$$

Where, *m* is the experimental value of H_2_ (mol); *n* is the reacted electron number (1); *F* is the Faraday's constant (96,500 C mol^−1^); *I* is the average current (A); *t* is the time (s).

## Results and discussion

### Structure and physical properties of the photoelectrode

**Figure 1:**
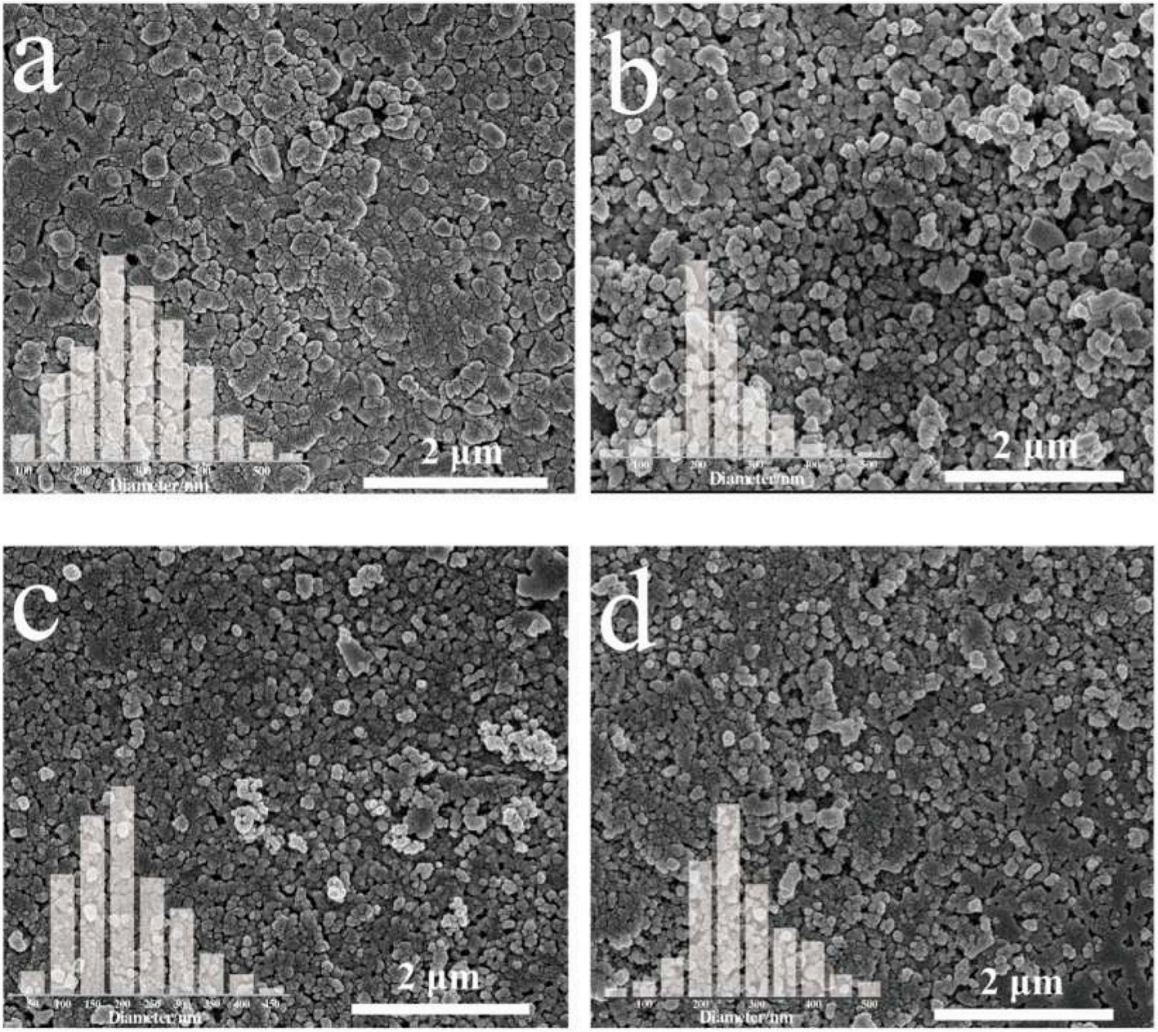
SEM images and particle range of **(a)** pure BiVO_4_; **(b)** BiVO_4_(Mo-1); **(c)** BiVO_4_(Mo-3); **(d)** BiVO_4_(Mo-5).

The SEM (Scanning Electron Microscopy) images of pristine BiVO_4_ and Mo-doped BiVO_4_ films are shown in Figure 1. All of the films present nanoparticle structure while a rougher, more disordered structure can be observed in the BiVO_4_ films. Besides, the incorporation of Mo could decrease the average size of BiVO_4_ with decreasing aggregation of the particles. The average diameter of pure BiVO_4_ is about 280 nm (Figure 1a), and the average diameter of Mo-doped BiVO_4_ are ranging from 200 to 250 nm, which are smaller than that of pure BiVO_4_ (Figures 1b–d). Besides, the smaller particle size of the as-prepared photocatalyst could provide more available active sites, which could be in favor of the PEC performance.

**Figure 2:**
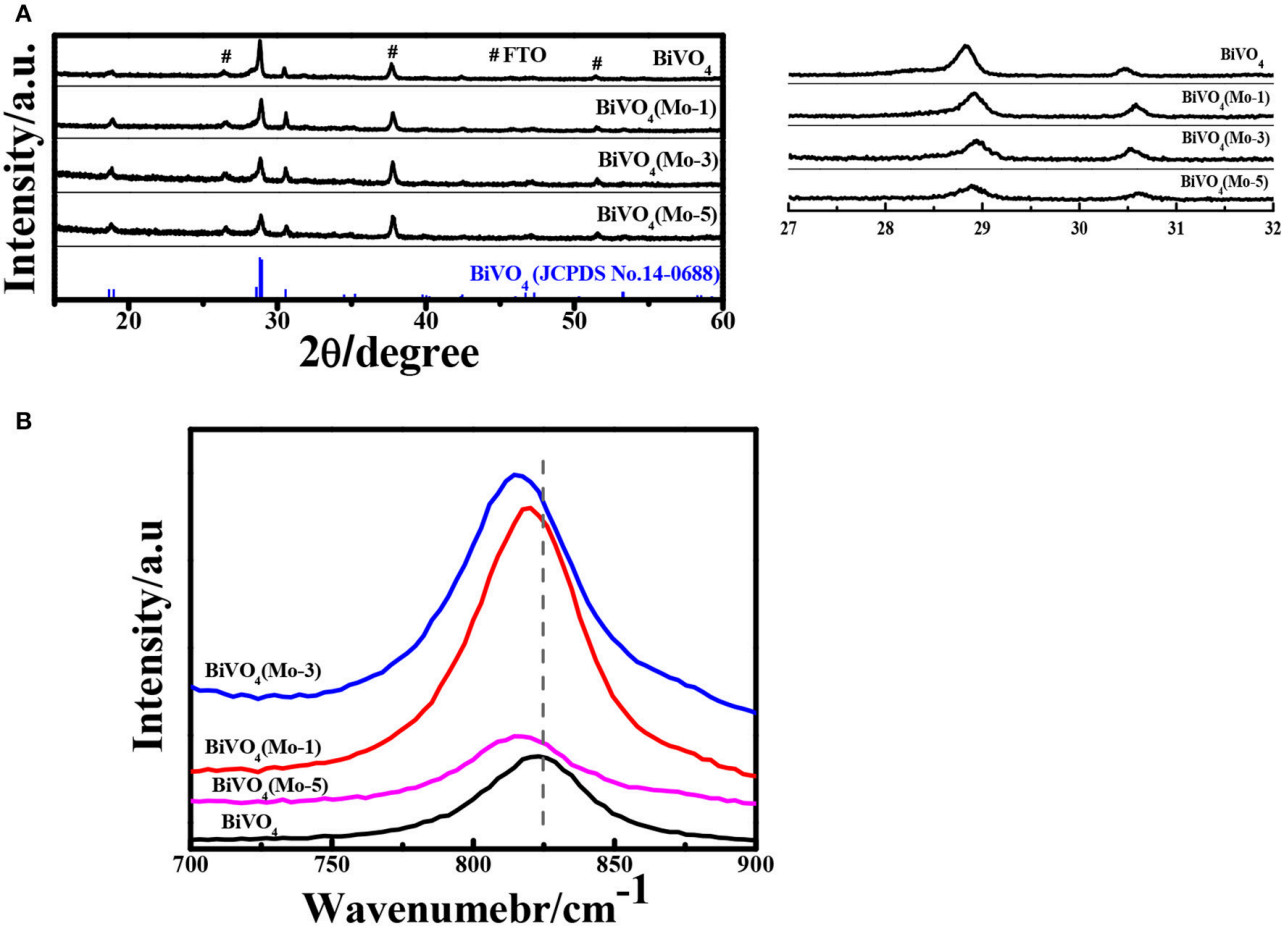
**(A)** XRD patterns; (#: FTO) **(B)** Raman spectra of pure BiVO_4_ and Mo doped BiVO_4_ with different concentration.

Figure 2A compares the XRD (X-Ray Diffraction) patterns of pure and Mo-doped BiVO_4_ films with different concentrations of Mo dopant. All the diffraction peaks are assigned to phase-pure monoclinic sheelite BiVO_4_ structure (JCPDS No. 14-0688). Since the films are very thin, the diffraction peaks of FTO substrate are also observed in XRD. No noticeable peaks of MoO_3_ is detected in the Mo-doped BiVO_4_, the results are consistent with previous study (Berglund et al., 2012; Luo et al., 2013; Chen et al., 2015; Nair et al., 2016; Thalluri et al., 2016). Additionally, the main peaks of the monoclinic BiVO_4_ structure shift to lower intensity and higher scattering angles in the Mo-doped BiVO_4_ films since the radius of Mo is higher than that of V. This represents a shrinkage or an enlargement of the *d* spacing of corresponding crystal planes due to incorporation of dopant cations into V sites of BiVO_4_ (Parmar et al., 2012). As the pure phase and modification in *d* spacing, it can be calculated that the Mo have been effectively incorporated into the crystal lattice of BiVO_4_ with the monoclinic phase unchanged (Parmar et al., 2012). Furthermore, the crystallite sizes calculated by using the Scherrer formula are 110, 117, 83, 85 nm for the BiVO_4_, BiVO_4_(Mo-1), BiVO_4_(Mo-3), BiVO_4_(Mo-5), respectively.

Though XRD shows some trace that Mo have been doped in the crystal of pure BiVO_4_. However, it is too rough to identify the doping sites in the crystal lattice because of the low doping concentration. To probe the doping sites and local distortions of Mo-doped BiVO_4_, the Raman spectra were measured and the results are shown in Figure 2B. The Raman mode located at 829 cm^−1^ is assigned to the symmetric stretching mode of ${\text{VO}}_{4}^{3-}$ units. It is clear that the symmetric stretching mode in Mo-doped BiVO_4_ shifts to a lower wave number, which suggests Mo^6+^ substitutes V^5+^ in the ${\text{VO}}_{4}^{3-}$ tetrahedron (Luo et al., 2013; Zhang et al., 2014).

**Figure 3:**
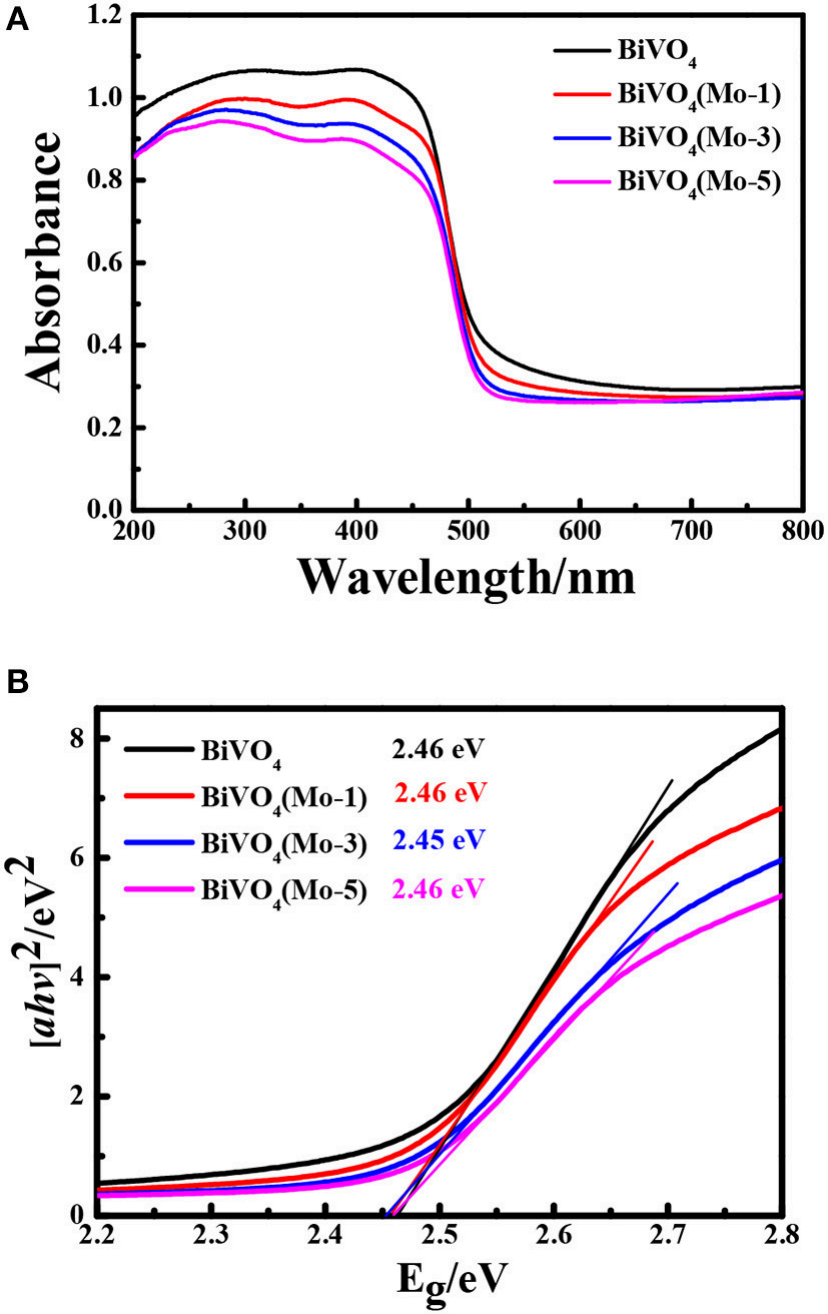
**(A)** UV-Vis DRS spectra and **(B)** Tauc plots of pure BiVO_4_ and Mo doped BiVO_4_ with different concentration.

Optical properties of the films are very important for the PEC performance. Figure 3A displays the UV-Vis DRS absorption spectra of the four films. All of the films present a strong absorption in the UV-Vis range, and the incorporation of Mo can hardly affect the absorption edges of BiVO_4_. Besides, there isn't any peak shift in the UV-Vis spectra of Mo-doped films, which indicates there is not existence of the phase transfer from monoclinic to tetragonal structures with doping of Mo (Figure 3A; Pattengale and Huang, 2016). As the pure BiVO_4_ shows a larger optical absorbance at wavelengths (>325 nm), thus the pure BiVO_4_ are less porous than the Mo-doped BiVO_4_ (Nair et al., 2016).

The optical band gap energy can be calculated by the following equation:

(4) $$\begin{array}{l} \alpha h\nu =A{\left(h\nu -E_{\text{g}}\right)}^{\text{n}/2} \end{array}$$

where, α, *h*ν, *A*, and E_g_ are the absorption coefficient, photo energy, constant and band gap energy, respectively. The value of n depends on whether the transition is direct (*n* = 1) or indirect (*n* = 4). The bandgap of all the films are about 2.5 eV (Figure 3B), which is consistent with the reported band gap of BiVO_4_ (Zhang et al., 2014). This result indicates that the incorporation of low amount of Mo could hardly influence the bandgap of BiVO_4_, and it is considered to be the characteristic band gap of monoclinic phase of BiVO_4_.

**Figure 4:**
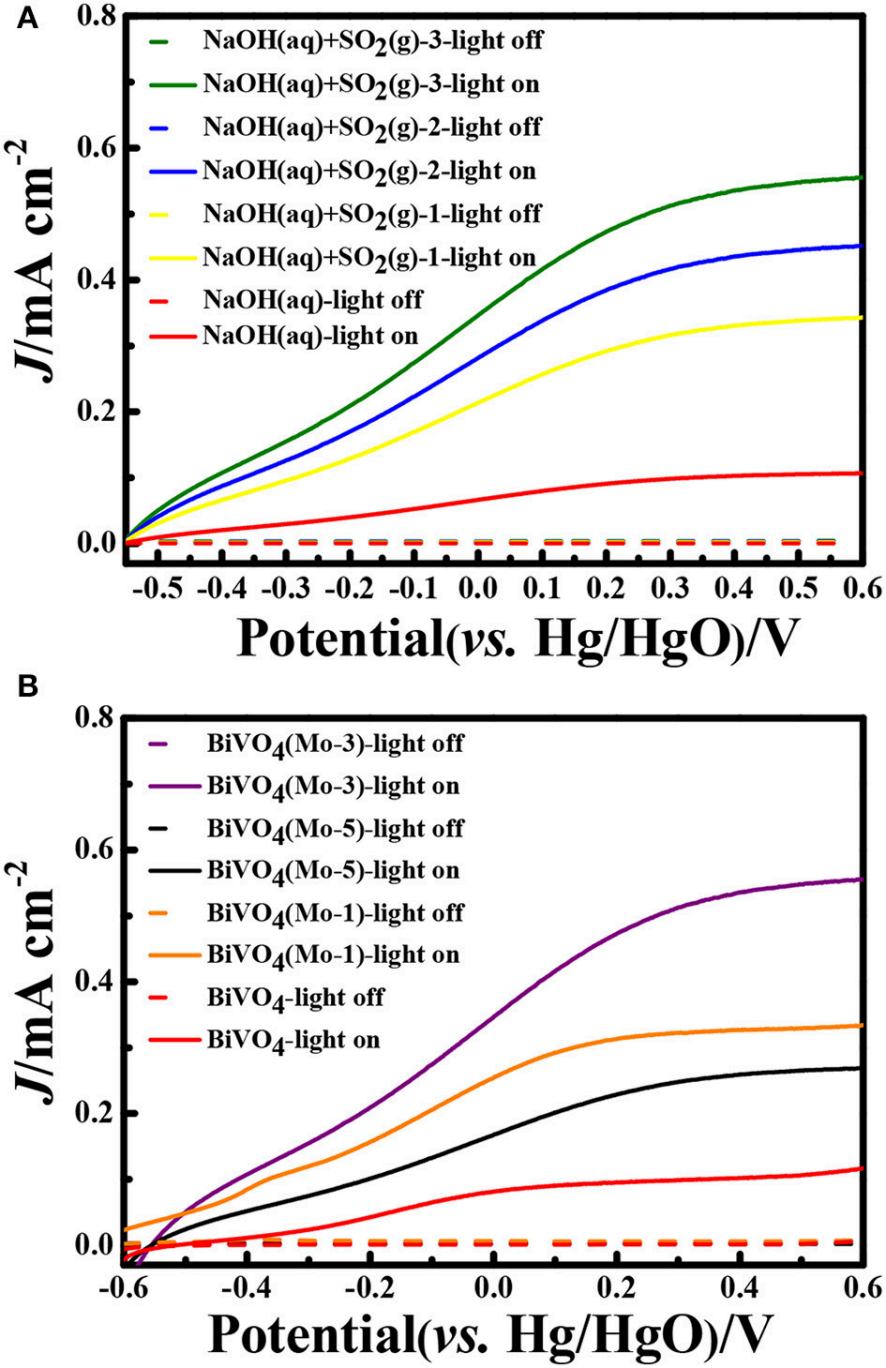
Linear Sweep Voltammograms (LSV) curves **(A)** BiVO_4_(Mo-3) in different electrolyte systems; **(B)** different photoanodes in NaOH(aq)+SO_2_(g)-3 electrolyte. (Scan speed: 10 mV s^−1^).

In the experiments, the SO_2_ gas was absorbed by NaOH solutions and the concentration of ${\text{SO}}_{3}^{2-}$ was detected. Through analyzing, the removal efficiencies of SO_2_ gas were about 98%, it indicated that the absorption method could removal SO_2_ completely. In order to determine the PEC properties of the photoelectrodes, the Linear Sweep Voltammograms (LSV) were measured both in dark and under AM 1.5 G illumination (100 mW cm^−2^; Figure 4). After analyzing the photocurrent densities of the photoanodes in different electrolyte systems after absorbing SO_2_ (Figure 4A), it is concluded: (1) the photocurrent densities are negligible under dark conditions in different electrolyte systems; (2) the photocurrent densities could be significantly enhanced after inletting SO_2_ into electrolyte. Since the introduction of SO_2_ into electrolyte, the concentration of ${\text{SO}}_{3}^{2-}$ could be increased, and the oxidation reaction of ${\text{SO}}_{3}^{2-}$ needs lower activation energy and kinetically faster than that of water, thus the formed ${\text{SO}}_{3}^{2-}$ consumes the photogenerated holes instantaneously and generates a higher photocurrent density than that of water. Though the doping of Mo could decrease the light-absorbing slightly (Figure 3A), but it could significantly enhance the bulk charge carrier transportation of BiVO_4_. Therefore, the PEC performance of Mo-doping BiVO_4_ is higher than that of BiVO_4_. The photocurrent density is greatly dependent on the amount of SO_2_ absorbed in the electrolyte. In NaOH(aq)+SO_2_(g)-3, the photocurrent density is improved by 1.2 times, 1.6 times, and 5 times than that of NaOH(aq)+SO_2_(g)-2, NaOH(aq)+SO_2_(g)-1, NaOH(aq) at 0.5 V vs. Hg/HgO, respectively. The photocurrent density is significantly improved by 5 times as compared with our previous research on porous BiVO_4_ (Han et al., 2017).

Compared with the four different electrode films in NaOH(aq)+SO_2_(g)-3 electrolyte, it is clear that BiVO_4_(Mo-3) displayed an outstanding PEC performance. The photocurrent density of BiVO_4_(Mo-3) is 1.7 times than that of BiVO_4_(Mo-1), 2 times than that of BiVO_4_(Mo-5), and 5 times than that of BiVO_4_ at 0.5 V vs. Hg/HgO (Figure 4B). The current densities of BiVO_4_(Mo-3) in NaOH(aq) with and without SO_2_(g)-3 at 0.6 V vs. Hg/HgO are ca. 0.6 and 0.1 mA cm^−2^, respectively; the current density in NaOH(aq)+SO_2_(g)-3 is 6 times higher than that in NaOH(aq). On the other hand, the evolution rate of H_2_ in NaOH(aq)+SO_2_(g)-3 (39.4 μmol h^−1^ cm^−2^) is more than 40 times higher than that in NaOH (0.92 μmol h^−1^ cm^−2^) in the Table 2. The above results indicate that the amount of Mo at 3 atom% could be an optimal choice.

**Table 2:** The current density, theoretical and experimental evolution rate of H_2_, and the Faradaic efficiency in different electrolytes [A−0.1 M NaOH; B—NaOH(aq)+SO_2_(g)-3].

**Items**	**BiVO**_**4**_		**BiVO**_**4**_**(Mo-1)**			**BiVO**_**4**_**(Mo-3)**			**BiVO**_**4**_**(Mo-5)**		
	**A**	**B**	**A**		**B**	**A**		**B**	**A**		**B**
	**L**	**L**	**D**	**L**	**L**	**D**	**L**	**L**	**D**	**L**	**L**
Current density/mA cm^−2^	0.01	0.8	0.005	0.03	1.85	0.007	0.05	2.1	0.004	0.023	1.6
Theoretical evolution rate of H_2_/μmol h^−1^ cm^−2^	0.19	14.8	0.092	0.55	34.2	0.13	0.92	39.4	0.075	0.43	29.6
Experimental evolution rate of H_2_/μmol h^−1^ cm^−2^	0.18	14.4	0.088	0.54	33.7	0.126	0.90	38.8	0.071	0.41	29.2
Faradaic efficiency/%	95	97	96	98	99	97	98	98	95	95	99

**Figure 5:**
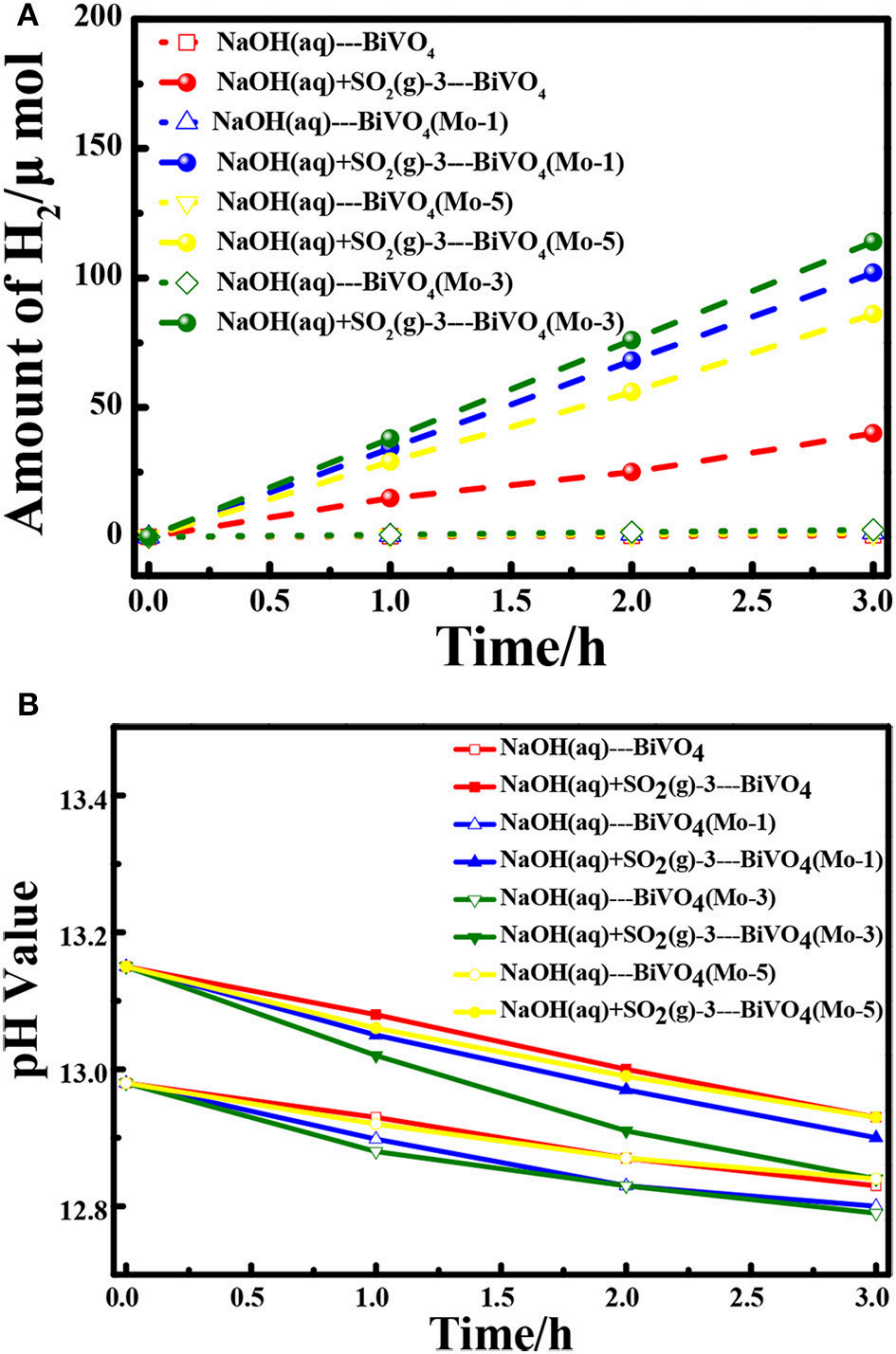
**(A)** Hydrogen generation at an applied potential of 1.6 V in two-electrode configuration for 3 h; **(B)** The variation of pH values of electrolytes during the process.

The experiments for H_2_ evolution in different systems were measured with a two-electrode configuration at a bias of 1.6 V under AM 1.5G irradiation. Figure 5A shows the amount of H_2_ evolved in different systems and Table 2 summarized the data of current densities, theoretical/ experimental evolution rates of H_2_ and the Faradaic efficiencies in the different systems. Comparing each photoanodes in different solution systems, it is clear that the SO_2_ removal could significantly facilitate the current density and evolution rate of H_2_, which is in good agreement with our previous research (Han et al., 2017). Besides, the current density and evolution rates for BiVO_4_(Mo-1, 3, 5) under light irradiation are higher than that under the dark condition. Furthermore, all of the Mo doped BiVO_4_ photoanodes show more attractive performance than that of pure BiVO_4_, and the BiVO_4_(Mo-3) performed the best H_2_ generation activities. As calculated, a highest H_2_ evolution rate of 39.4 μmol h^−1^ cm^−2^ is realized in NaOH(aq)+SO_2_(g)-3 with BiVO_4_(Mo-3) as photoanode, and the H_2_ evolution rate is only 0.19 μmol h^−1^ cm^−2^ in NaOH(aq) with the BiVO_4_ as photoanode, suggesting the H_2_ production can be enhanced about 200 times with the removal of SO_2_ simultaneously with 3 atom% Mo-doped BiVO_4_. Furthermore, the theoretical evolution rates of H_2_ are close to theoretical rates in each system, indicating a high Faradaic efficiency of H_2_ production (higher than 95%). Besides, the catalysts could not dissolve although the solution is a strong basic aqueous solution.

**Figure 6:**
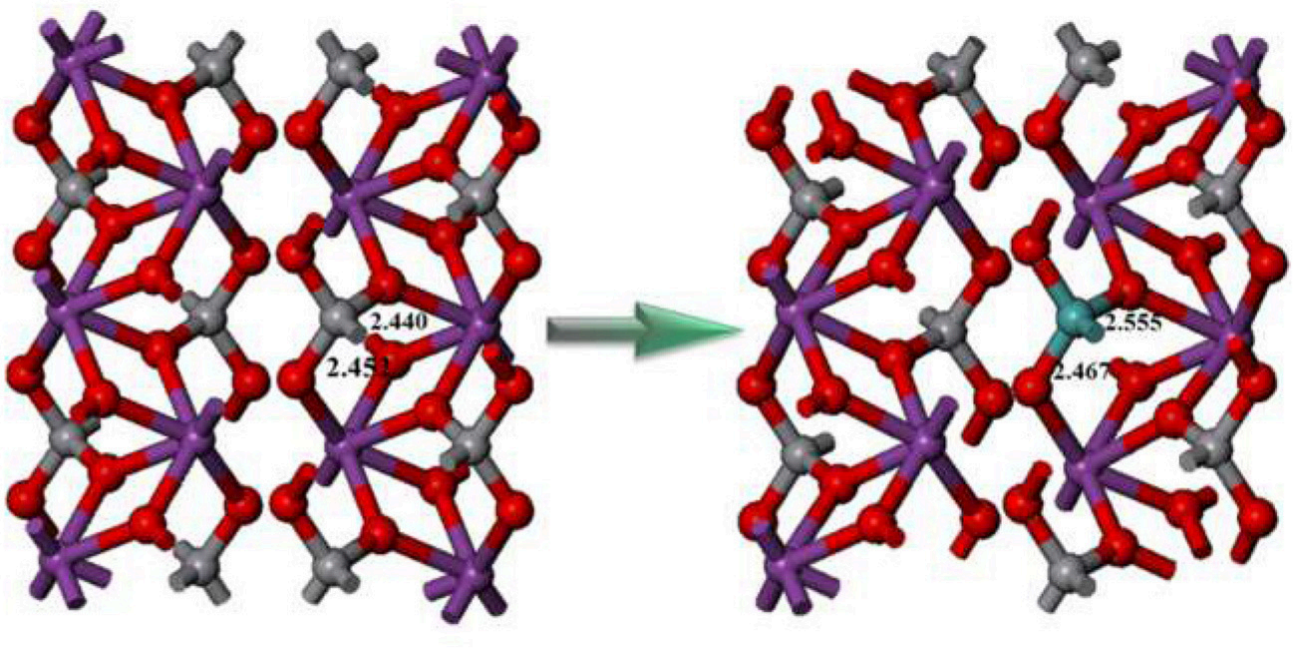
The conventional cell of pure monoclinic sheelite BiVO_4_ **(left)** and supercell of Mo doped monoclinic sheelite BiVO_4_ **(right)**.

In order to better understand the structure reconstruction with/without Mo doping, the geometric structures of pristine and the Mo-doped BiVO_4_ were compared in Figure 6. It is clear that the doping of Mo could increase the length of Bi-O bonds. This phenomenon indicates that the original coordinated O atoms are “shifted” toward the doped Mo sites, and introduce “oxygen vacancies.” As well-known, the oxygen vacancies could play an important role in improving the photocatalytic performance. Besides, the Mo^6+^ ion substitute the V site in monoclinic sheelite BiVO_4_ could improve the transportation of photoinduced carriers, which could facilitate the charge carrier separation and leads to suppressed bulk recombination (Ding et al., 2014). Therefore, the photocatalytic activity is enhanced significantly after Mo-doping, as shown in Figure 4.

**Figure 7:**
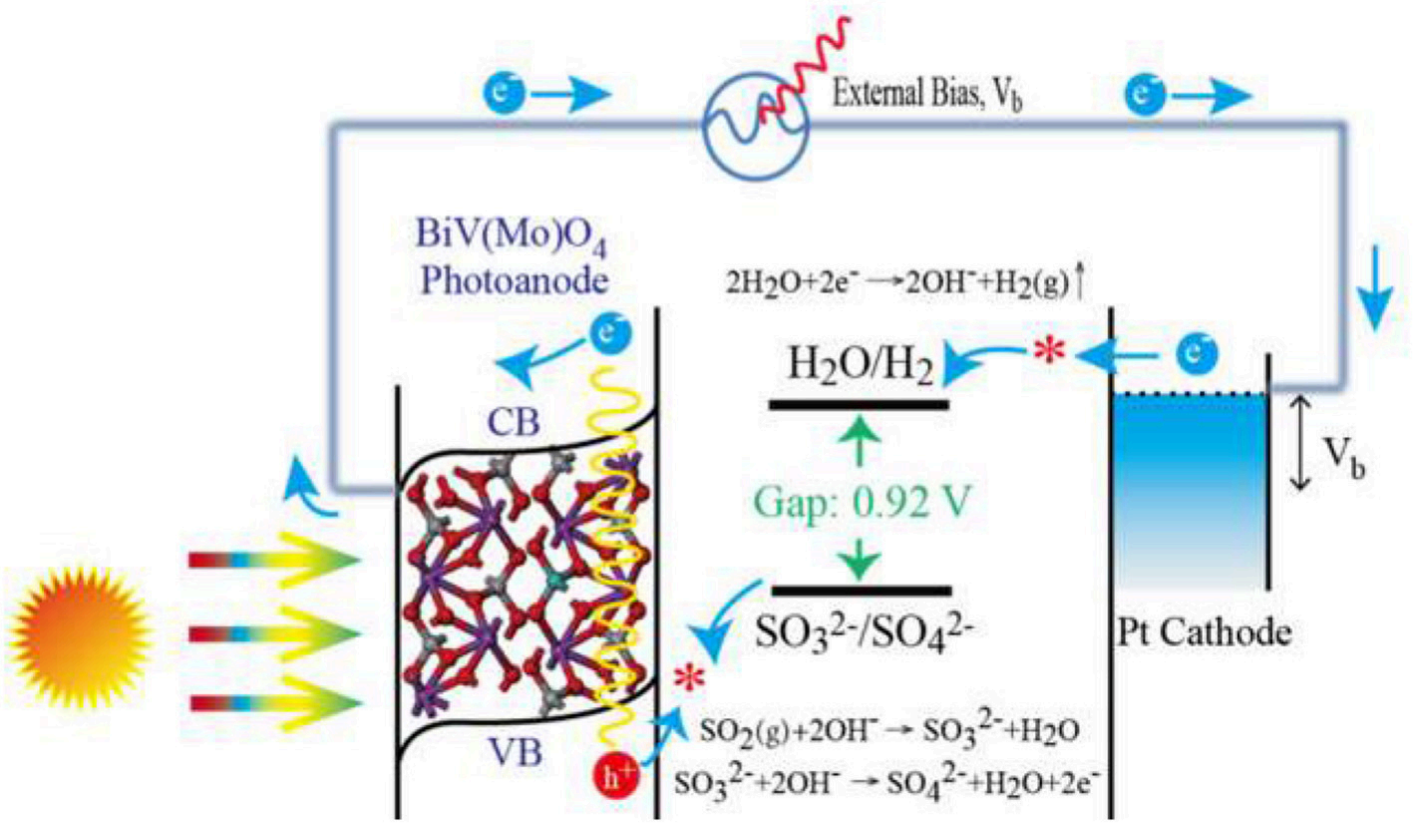
Schematic illustration of H_2_ generation during the PEC process with SO_2_ removal.

With the existence of flue gas SO_2_ removal, ${\text{SO}}_{3}^{2-}$ was formed in electrolyte solution. Then the photo-generated holes could be more quickly consumed by the formed ${\text{SO}}_{3}^{2-}$ than that without ${\text{SO}}_{3}^{2-}$ (pure NaOH solution), because oxidation of ${\text{SO}}_{3}^{2-}$ has a much lower activation energy and kinetically much faster than water oxidation or OH^−^ oxidation (McDonald and Choi, 2012; Seabold and Choi, 2012). The oxidation of SO_2_ is thus proposed to replace the oxygen evolution reaction in water splitting, which can improve the water splitting efficiency and reduce the cost of H_2_ production. The whole process for SO_2_ removal with simultaneous production of H_2_ is thus summarized and illustrated in Figure 7. Mo-doped BiVO_4_ is first excited to generate *e*^−^ in conduction band (CB) and create hole (*h*^+^) in the valence band (VB) at the same time. With the assistance of space charge layer and extra bias, the *e*^−^ transfer to the cathode and participate in cathode reaction for H_2_ production. While the formed of ${\text{SO}}_{3}^{2-}$ after flue gas SO_2_ absorption is oxidized by the holes on the photoanode. As the Mo incorporated in BiVO_4_ could improve the electronic conductivity of pure BiVO_4_ (Luo et al., 2011; Pilli et al., 2011), therefore, the *e*^−^ produced in Mo doped BiVO_4_ moves faster than that produced in BiVO_4_, and shows a significantly increased photocurrent density (Figure 4) and rate of H_2_ production (Figure 5). The reactions are summarized as following:

(5) $${\text{SO}}_{2}(\text{g})+{\text{OH}}^{-}\to {\text{HSO}}_{3}^{-}$$

(6) $${\text{HSO}}_{3}^{-}+{\text{OH}}^{-}\to {\text{SO}}_{3}^{2-}+{\text{H}}_{2}\text{O}$$

Reaction on photoanode (Mo-doped BiVO_4_ film):

(7) $${\text{SO}}_{3}^{2-}+2{\text{OH}}^{-}\to {\text{SO}}_{4}^{2-}+{\text{H}}_{2}\text{O}+2e^{-}$$

Reaction on cathode (Pt wire):

(8) $$\begin{array}{l} 2{\text{H}}_{2}\text{O}+2{\text{e}}^{-}\to {\text{H}}_{2}\left(\text{g}\right)+2\text{O}{\text{H}}^{-} \end{array}$$

The total reaction:

(9) $$\begin{array}{l} \text{S}{\text{O}}_{2}\left(\text{g}\right)+2\text{O}{\text{H}}^{-}\overset{\text{hv}}{\to }{\text{H}}_{2}\left(\text{g}\right)+\text{S}{\text{O}}_{4}^{2-} \end{array}$$

## Conclusions

In summary, the effect of Mo doping on BiVO_4_ used as photoanode for H_2_ production with simultaneously flue gas SO_2_ removal is investigated. The 3 atom% Mo-doped BiVO_4_ (BiVO_4_(Mo-3)) possessed the best PEC activity due to its better charge carrier transportation. With the help of Mo, the H_2_ evolution rate and SO_2_ removal rate of Mo-doped BiVO_4_ almost 3 times higher than pristine BiVO_4_. Through this process, the SO_2_ in flue gas is removed and collected to produce H_2_, which could greatly reduce the cost of desulfurization process, and even make it profitable.

## Author contributions

JH: assisted in design of the experiments and wrote the manuscript; KL: performed the experiments and wrote the manuscript; HC: assisted in the analysis and interpretation of the data; LZ: planned the project, designed the experiments, and also wrote the manuscript.

### Conflict of interest statement

The authors declare that the research was conducted in the absence of any commercial or financial relationships that could be construed as a potential conflict of interest. The reviewer, GJ, and handling Editor declared their shared affiliation.
